# Reactions to treatment debriefing among the participants of a placebo controlled trial

**DOI:** 10.1186/1472-6963-5-30

**Published:** 2005-04-22

**Authors:** Zelda Di Blasi, Fay Crawford, Colin Bradley, Jos Kleijnen

**Affiliations:** 1 Health Psychology Program, Laurel Heights Campus, University of California San Francisco, 3333 California Street, Suite 465, UCSF Box 0848, CA 94143-0848, USA; 2 Osher Center for Integrative Medicine, University of California San Francisco, 1701 Divisadero Street, UCSF Box 1726, CA 94143-1726, USA; 3 Dental Health Services Research Unit, Dundee, DD1 4HR, UK; 4 Department of General Practice University College Cork, Cork, Ireland; 5 Centre for Reviews and Dissemination, University of York, York YO10 5DD, UK

## Abstract

**Background:**

A significant proportion of trial participants respond to placebos for a variety of conditions. Despite the common conduct of these trials and the strong emphasis placed on informed consent, very little is known about informing participants about their individual treatment allocation at trial closure. This study aims to address this gap in the literature by exploring treatment beliefs and reactions to feedback about treatment allocation in the participants of a placebo-controlled randomized clinical trial (RCT).

**Methods:**

Survey of trial participants using a semi-structured questionnaire including close and open-ended questions administered as telephone interviews and postal questionnaires. Trial participants were enrolled in a double-blind placebo-controlled RCT evaluating the effectiveness of corticosteroid for heel pain (ISRCTN36539116). The trial had closed and participants remained blind to treatment allocation. We assessed treatment expectations, the percentage of participants who wanted to be informed about their treatment allocation, their ability to guess and reactions to debriefing.

**Results:**

Forty-six (73%) contactable participants responded to our survey. Forty-two were eligible (four participants with bilateral disease were excluded as they had received both treatments). Most (79%) participants did not have any expectations prior to receiving treatment, but many 'hoped' that something would help. Reasons for not having high expectations included the experimental nature of their care and possibility that they may get a placebo. Participants were hopeful because their pain was so severe and because they trusted the staff and services. Most (83%) wanted to be informed about their treatment allocation and study results. Over half (55%) said they could not guess which treatment they had been randomized to, and many of those who attempted a guess were incorrect. Reactions to treatment debriefing were generally positive, including in placebo responders.

**Conclusion:**

Our study suggests that most trial participants want to be informed about their treatment allocation and trial results. Further research is required to develop measure of hope and expectancy and to rigorously evaluate the effects of debriefing prospectively.

## Background

One of the most famous examples of the placebo effect is an account given by Dr. Klopfer of a trial patient with advanced cancer randomized to a new drug called 'Kebriozen'. Klopfer describes how within ten days: 'all signs of his disease [had] vanished'. The patient relapsed within two months when he learned that trials results were inconclusive. His clinician somehow managed to convince the patient that he had: 'a new super refined, double strength product', but instead administered saline injections. The patients' response to these injections was described as: 'even more dramatic than the first'. He remained symptom free for over two months, until he read that: "nationwide tests show Krebiozen to be a worthless drug in treatment of cancer". Within a few days of this report, Mr. Wright was readmitted to the hospital in extremis. His faith was now gone, his last hope vanished, and he succumbed in less than two days' [1].

Much has been written on the ethics of obtaining informed consent from trial participants [2] and the ethics of using placebos in clinical trials [3,4]. However, the ethics of debriefing ('disclosing', 'unmasking', 'unblinding'), at study closure has been overlooked. Debriefing consists of informing trial participants about their individual treatment allocation and study results. Research in this area is scarce, but it is of particular relevance for placebo-controlled trial participants.

There is some evidence that these participants may be kept in the dark about their allocation once the experiment is over. In a survey published in the *British Medical Journal*, we found that less than half of trial investigators informed participants of placebo-controlled trials about their treatment allocation at trial closure [5]. The most common reason for continuing to keep patients 'blind' was that investigators had: 'never considered the option of informing patients'. This is despite government standards for research emphasizing the need to debrief and share study findings with participants [6].

Debriefing shows respect and appreciation to trial participants and it symbolises investigators' desire to involve patients and share the knowledge gathered during the experiment. However, debriefing placebo responders can also be disruptive. Unless information is disclosed sensitively and effectively (e.g. in the context of trial results and explaining what is understood to trigger a placebo effect), healing reactions in placebo responders may be broken. In one trial[7], when placebo responders were told that the treatment allocated to them had been a placebo, most of them relapsed and had to be prescribed the 'real' medication[8]. In a separate trial, though fetal-cell implantation was found to be as effective as sham surgery for Parkinson patients, most of the placebo responders still wanted to receive the implants once treatment allocation was unblinded a year later[9]. When patients who had received the sham surgery were told that they could not receive the real but now 'discredited' surgery they had been promised during the informed consent stage, 70% were disappointed and 'outraged' because of the dramatic effects they had already received from the sham surgery[10]. These findings suggest that just like patients who think they have received an active treatment can have significant physiological and psychological improvements[11], patients who learn they have actually been deceived with a sham treatment can worsen.

Our knowledge around treatment debriefing in the participants of placebo-controlled trials is scarce and based on a handful of reports. In this study we set out to fill this gap in the literature by: 1) exploring treatment expectations; 2) ability to guess treatment allocation; 3) wish to be debriefed and 4) reactions to unmasking in the participants of a placebo-controlled RCT testing the effectiveness of corticosteroid for heel pain.

Plantar heel pain is a common painful condition where placebo effects have been shown to exist[12]. A systematic review of interventions for the management of the painful heel was unable to find compelling evidence of effectiveness for any of the therapies evaluated in RCTs. While various interventions are used to treat it, including steroid injections, there is limited evidence for their effectiveness[13,14]. In an RCT evaluating the effectiveness of a corticosteroid injection in the treatment of plantar heel pain, one of us found that corticosteroid was effective at one month, but not at three and six months[15], and participants were not informed about their treatment allocation at study closure. The trial was conducted between January 1995 and December 1998 at the Center for Rheumatology, University College London.

The control used in this trial was a local anaesthetic. This is an active drug, but because is is assumed to be ineffective for plantar heel pain, it was used as a credible placebo in this trial. Anaesthetic effects are short-term (5–6 hours), and the earliest evaluation of a therapeutic effect following treatment in this trial was at 1 month. Its effects are therefore thought to be 'non-specific'. Not least of all because at the time the outcomes were collected local anaesthetic would be pharmacologically inert (i.e. the effect of numbness would have worn off).

## Methods

**Figure 1:**
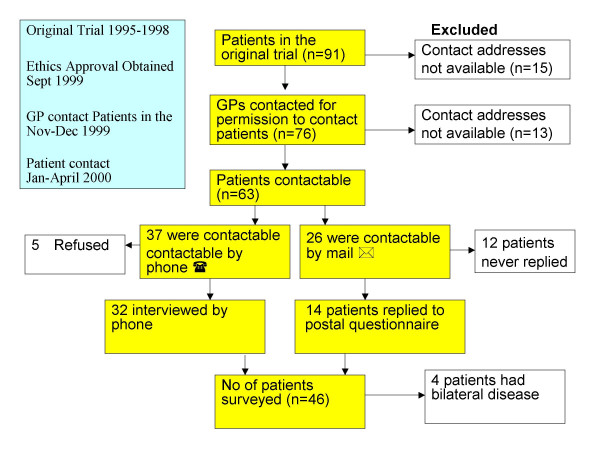
Flowchart of recruitment process

Patient and GP contact details were obtained from the hospital database of the original trial. The boxes (Fig. 1) illustrate the patient recruitment process.

Ethics approval was received from University College London ethics committee. Following guidance and ethical approval we wrote one letter and used two follow-up reminders to seek the consent of the trial patients' GPs for us to re-establish contact with them. For GPs who gave permission, a letter was written to each patient inviting them to share their experiences of the treatment received and satisfaction with the care provided for their heel pain.

Telephone contact was made by the first author (ZDB) and data gathered from January 2000 to April 2000, using a semi-structured questionnaire developed in collaboration with two chartered health psychologists.

Participants were asked general questions about their heel pain (e.g. beliefs about possible causes), and open questions about what they remember of the study and the doctor who treated them. We asked closed questions to assess:

(i) Treatment expectations ("*Before you were given the injection, did you have any expectations about how effective it would be*?", "*Were your expectations: 'very high', 'somewhat high', 'not sure', or 'low"'*?);

(ii) Ability to guess treatment allocation ("*What treatment do you think you got*?', '*How confident are you that you got X"*? with responses ranging from: '*very confident' *to '*not at all confident'*);

(iii) Wish to be debriefed ("*Would you like to know what you got*?", "*Would you like to know the overall study results*?").

Recordings of reaction to debriefing about treatment allocation and study results were left open. Responses were not tape recorded, but typed in shorthand and transcribed directly on a computer file. Patients who could not be contacted by phone were mailed a letter and a postal questionnaire, asking them to include a telephone number if they wanted to be contacted about their treatment allocation.

### Analysis

Narrative descriptions were integrated with the quantitative data derived from the original trial. Patients who had a lower pain score from baseline to one month and who had been allocated to the local anaesthetic were described as 'placebo responders' (PR). Those who did not respond at one month were described as 'placebo non responders' (PNR). Participants who had a lower pain score one month after treatment and who had been allocated to the steroid arm were described as 'steroid responders' (SR), while those who didn't respond to the steroid at one month were described as 'non responders' (SNR). The main outcome was pain was assessed using a 10 cm Visual Analogue Scale (VAS), where '0' indicated no pain and '10' indicated worst pain imaginable.

## Results

Contact details of 76 of the original 91 GPs and patients were available. GPs notified us not to contact 13 of the 76 patients (1 deceased, 1 terminally ill, 1 under investigation, 6 had moved away, no records for 2, no reason given for 2). A total of 63 patients were invited to take part in our study. All patients for whom we had a telephone number were telephoned. Of these, 32 patients agreed to be interviewed and five refused (2 were sick and 1 was caring for a sick person; 2 did not give a reason). Twenty-six participants were not contactable by telephone as their number was not obtainable (6), it was ex-directory (2), wrong (6), or always engaged (5), four had no phone and three had moved away. In order to increase our response rate, postal questionnaires were mailed to the 26 patients who were not contactable by telephone. Fourteen of the 26 (54%) patients responded to our postal questionnaire. A total of 73% contactable individuals (46 of 63) agreed to participate in our survey.

In the original trial, treatments were randomized to *episodes of heel pain*, rather than *per patient*. There were 91 patients with 106 episodes of heel pain. Seven of these patients had bi-lateral heel pain and it was possible for these patients to receive both steroid and placebo injection, or 2 steroid injections, or 2 placebo injections. These individuals were excluded from this survey.

A total of 53 episodes of pain were randomized to *placebo *and 53 were randomized to *steroid*. Corticosteroid was found to be significantly more effective than the local anaesthetic at one month, but not at three or at six months. Twenty-eight percent of the entire trial population still had heel pain at the end of the trial.

In the present follow-up we contacted 46 patients with 49 episodes of pain (51% of the original trial). Of these, four patients were excluded because they had bilateral disease. Our total sample consisted of 42 participants. Of these, 24 patients had been randomized to receive a steroid injection and 18 had been randomly allocated to receive a local anaesthetic, or placebo. Eighteen of 24 (75%) patients who were randomized to the steroid injection responded (SR), and 7 of 18 (39%) of patients who were randomized to the placebo responded (PR). Six participants who received a steroid injection failed to respond (SNR) and 11 participants who received a local anaesthetic did not respond (PNR). A detailed description and analysis of pain scores is provided in a previous publication from the original trial[16].

### 1. Participants' expectations about treatment

Trial participants were asked whether they had any expectations about the effectiveness of the treatment, prior to receiving this. Most (79%) said they did not have any expectations prior to receiving treatment, but 'hoped' something would help. For this reason we asked participants how hopeful they were that the treatment would help them (see Table 1).

**Table 1:** Treatment Expectations in trial participants

	**PR n (%)**	**PNR n (%)**	**SR n (%)**	**SNR n (%)**	**TOTAL n (%)**
Very Hopeful	-	3 (27%)	7 (39%)	1 (17%)	11 (26%)
Hopeful	3 (43%)	4 (36%)	2 (11%)	2 (33%)	11 (26%)
Not sure	4 (36%)	4 (36%)	6 (33%)	2 (33%)	16 (38%)
Not hopeful	-	-	3 (17%)	1 (17%)	4 (9%)

PR: Placebo Responder; PNR: Placebo Non Responder; SR: Steroid Responder: SNR: Steroid Non Responder

A total of 38% (16 of 42) said they weren't sure about how effective the treatment would be prior to receiving this. Four participants explained that they didn't know what to expect because of the artificial nature of their care ("*As far as I remember the treatment was coded so that they could not be immediately identifiable"*, "*No idea, I wasn't told"*, "*I knew it was an experiment and there was no way of knowing that it would help", "50/50 because there was no guarantee that it would cure it"*).

About half of the participants (52%, 22 of 42) said they were either 'very hopeful' or 'hopeful' (e.g. "*I didn't have any expectations, I hoped something would help"*). Reasons for being hopeful included the fact that the pain was so severe ("*Very hopeful because of the pain, it was extraordinary that it just came and stayed like that"*, "*You really hope because the pain is so bad, and it can be quite disappointing", "Hopeful, I really wanted to continue with my life without the pain I was experiencing"*), the credibility of the staff ("*Very hopeful ... they seemed to be very efficient; they seemed to know what they were doing"*), and trust in the health services ("*I thought it would cure it, I felt definite. I have a feeling that if I go to hospital they are going to do their utmost and will do their best so I am quite happy and relaxed about that"*). Another participant said she was open to give the treatment a try (e.g. "*I felt I would give it a try as I had already tried acupuncture and massage and they didn't help. I knew it was a treatment and that it was part of a trial, but I was happy to give it a go"*).

Nine percent (4 of 42) said they were not hopeful, three of which were steroid responders. Reasons for not being hopeful included previous experience with healthcare ("*Not very hopeful. Normally when you go to hospital you have to return a few times"*).

Three participants commented on the fact that they did not expect the injection to be so painful ("*didn't really have any expectations ... I didn't know how painful it would be but it was terrible. I have had worse, but it was bad'*, "*The injection was more painful than I had expected and I am used to injections", "Having the injection was quite painful, more than I expected"*).

### 2. Guessing treatment allocation

When asked what treatment they thought they got, just over half (22 of 42, 55%) said they didn't know. Only 7 of the 19 (37%) participants who attempted a guess were correct about their treatment allocation, four were steroid responders (Table 2).

**Table 2:** Beliefs about Treatment

	**PR n (%)**	**PNR n (%)**	**SR n (%)**	**SNR n (%)**	**TOTAL n (%)**
It was a 'placebo' or an anaesthetic	------	2 (18%)	3 (17%) **X**	1 (17%) **X**	6 (14%)
It was a steroid or the 'real' thing	2 (29%) **X**	6 (54%) **X**	4 (22%)	1 (17%)	13 (31%)
I don't know	5 (71%)	3 (27%)	11 (61%)	4 (67%)	23 (55%)

PR: Placebo Responder; PNR: Placebo Non Responder; SR: Steroid Responder: SNR: Steroid Non Responder; Correct answer:  Incorrect answer:**X**

The local anaesthetic or placebo was described as: "*a dummy"*, "*the wrong one"*, "*the one with nothing in it"*, "*plain water*", and *"the one that wasn't going to work"*, while the active treatment was described more positively as: "*a new treatment"*, "*a new formula"*, "*the right one"*, the "*pain killer"*, and the "*real one"*.

One of the participants said he tried to guess what treatment the therapist was administering by looking into his eyes but he was still unable to break the blind ("*I did look into his eyes carefully to see what it was he was giving me. He looked interested and pleased to see I wasn't in pain, but I suppose that is because he is a doctor, he is happy when patients get better*").

A placebo responder explained that he did not know what he got because he felt that both treatments could be effective ("*Whether it was faith or a chemical interaction, I don't know, but it was effective. You can't be sure of anything, you have to leave the possibilities"*) and a steroid responder said he trusted in natural self-healing responses ("*I think some things go away by themselves, you just give the injection and it doesn't matter what's in it. I know these things. I am a GP. My hunch is that it was only a local"*). When asked what treatment he got, a steroid participant confidently guessed that this was a placebo, attributing his improvement to relaxation ("*An anaesthetic, I'm confident. I think that after the pain I was more relaxed and the pain wasn't as bad. When I am nervous and I am thinking about the pain it's worse and it comes back"*).

### 3. Wish to be debriefed

Most (83%, 35 of 42) trial participants wanted to know what treatment had been allocated to them (see Table 3).

**Table 3:** Wish to be debriefed

	**PR n (%)**	**PNR n (%)**	**SR n (%)**	**SNR n (%)**	**TOTAL n (%)**
Yes	6 (86%)	9 (82%)	16 (89%)	4 (67%)	35 (83%)
No	-	2 (18%)	2 (11%)	1 (17%)	5 (12%)
Inappropriate	1 (14%)	-	-	1 (17%)	2 (5%)

PR: Placebo Responder; PNR: Placebo Non Responder; SR: Steroid Responder: SNR: Steroid Non Responder

One participant was upset that he had never been debriefed: "*They should have written to everyone ... they are obliged to inform patients. Patients are treated almost as children ... they [investigators] just want to know about their experiments*".

Five participants preferred not to be debriefed (e.g. *"I am fine now"*). One participant was very upset because of negative experiences following the injection and another insisted that he had not participated in the study ("*They just went with the corticosteroid with me, I never had the option of the other on"*). These two participants were not debriefed.

All participants who wanted to be debriefed about their treatment allocation, also wanted to be informed about study results. Participants were told that: "*Corticosteroid was found to be significantly more effective than local anaesthetic at one month, but not at three and at six months"*.

### 4. Reactions to treatment debriefing

Reactions to debriefing in placebo responders ranged between slight embarrassment ("*Really? That makes me feel really silly, oh my God! ... I am cringing now") *to amazement and excitement ("*That is fantastic. That is a discovery! Human chemistry is the most effective of them all. I am really thrilled to hear I was given a local anaesthetic ... It is the faith, the trust we put in people*").

There was a similar variation among active treatment responders, between those who were thrilled to hear they got better thanks to a 'real' drug ("*It was? They couldn't fool me*!"), to those who believed that healing results spontaneously and incorrectly guessed that they had received a placebo, and were surprised to hear they had been injected with corticosteroid ("*It was? Oh, there you go...*").

Two placebo non-responders incorrectly guessed the treatment allocated to them had been the active treatment. When they were debriefed, both as well as a placebo non responder attributed their improvement to physiotherapy ("*The physiotherapy helped a lot"*, "*The physiotherapy was lovely, I could have managed without the injection, so I can't really say which was more effective"*, "*Oh right, I did have it (heel pain) after the injection, but they it started going after the physiotherapy, I suddenly woke up one morning, and I was able to put it (the foot) down"*).

Many of the reactions to study results tended to be exclamations ("*Mmmmm"*, "*Right"*, "*Ahhh"*, "*Ohh"*, "*Fair enough"*, "*Interesting"*). Three participants were very pleased to hear the study results ("*I am delighted to find out about the treatment and results. I am very satisfied with the study"*, *"These are really interesting findings"*, *"I think these (results) are right!*").

Three asked to find out more information about the study ("*Interesting, I would really like to read more about it"*, "I *would really like to read the study, would you mind sending me a copy? So you are doing a study of the study, that's interesting", "I am always interested in research, please send me a copy of the paper"*).

One participant asked what have we learned from this study and where do we go from here. She suggested that there should be more information for heel pain patients about the causes of heel pain and guidelines to help practice self-care.

## Discussion

In our study, most (79%, 33 of 42) trial participants said they did not have any 'expectations' prior to receiving treatment, but rather 'hoped' that something would help. This finding was surprising, considering that treatment expectations are considered to be one of the principle mechanisms of placebo effects [17-19].

We were not able to find a satisfying definition of 'hope' as distinct from 'expectation'. Both tend to be described as beliefs that a desired outcome will occur, with hopes often having an element of expectation[19]. Common use of the concepts would suggest that hopes are accompanied with a lesser degree of certainty than expectation, perhaps allowing for the possibility of dealing with disappointment. One of the participants in our survey explained how "*You really hope because the pain is so bad, and it can be quite disappointing"*.

Our findings are supported by those of a recent qualitative and in depth study examining expectations in the participants of a placebo-controlled RCT [20]. The team found that only one of the nine participants expected to experience significant improvements, most hoped they would achieve some benefit, and the rest worried about possible drug side-effects rather than anticipating improvement. The authors also determined that expectations changed as a result of self-monitoring, a finding that is in line with Lundh's placebo effect theory [21]. Lundh suggested that when individuals believe that a treatment will cure them they will selectively attend to signs of improvement, and attribute any improvements to the treatment.

The therapeutic role of hope has not been subject of scientific scrutiny, whereas the effects of expectancies have been much more under investigation, especially in the placebo effect literature [17,18,22]. It is useful to be aware of the difference between outcome expectancies, which are used to refer to consequences that follow actions and self-efficacy expectancies, or beliefs that one can successfully perform the actions required to achieve valued outcomes [23,24]. A third related construct is that of optimism, which has been defined as a generalized expectancy that one will experience good outcomes in life. While these constructs overlap in that they emphasize a belief that a desired outcome will occur in the future, they have been found to be separate. This distinction has been shown to be both general and robust across contexts, although "*All are related by the central core of expectancies" *(p.18) [19].

These findings point to the broader, multi-dimensional and dynamic nature of expectations, something which often fails to be considered in clinical research. In a systematic review of placebo controlled RCT's examining the therapeutic effects of treatment expectations [25], we found that expectations were measured using single item scales administered at one point in time, thus failing to capture the complex nature of expectations.

In our survey, when asked about what treatment they thought they had been randomized to more than half of the participants (55%, 22 of 42) hesitated to answer, perhaps afraid to appear foolish due to the negative connotations associated with placebos. Only 37% of the participants who volunteered to take a guess were able to correctly identify their treatment allocation, suggesting that the internal validity of the trial findings were unaffected by a breech in the trial blinding.

Most wanted to be informed about their treatment allocation and about study results and five individuals refused to be informed. Reactions to treatment debriefing varied between surprise, embarrassment, excitement, and attribution to another treatment, typically physiotherapy, to explain their recovery or improvement. Participants were generally interested to hear about the study findings, and three specifically asked to receive any published material from the study.

We found that participants varied in their understanding of the study and the treatments being investigated. Some were very clear about what was being investigated and the principles of randomization, while some did not realize they had been recruited into a study.

These findings are in line with a review which found that trial participants often have difficulty understanding the concept and purpose of a trial, randomization and double-blind procedure [26].

## Limitations of the study

Our study is limited by its retrospective nature and the time that elapsed since the original trial was conducted. The time lapse was at least a year, depending on the stage in which participants may have dropped out during the trial. The delay largely resulted from difficulties in obtaining ethical approval, which took nine months. The time lapse and the elderly age of these patients may have been affected patients' recall. Although many trial participants could not remember the names of the treatments being investigated, most were able to distinguish between a pharmacologically active and a non-specific treatment which they described in their own words as placebo (e.g. 'the one with nothing in it') or the wrong treatment.

The trial is the largest RCT examining the effectiveness of corticosteroid for heel pain to date. Despite this, the trial population we were able to follow up was relatively small. Furthermore, 43% of the participants interviewed reported that their heel pain was recurrent and for almost 20% it was continuous. The cyclical nature of heel pain further limits our ability to extrapolate on the relationship between cognitions and outcomes.

Reactions to debriefing were recorded immediately after the information was communicated. While this method identified immediate response to debriefing, research would benefit from monitoring more long-term effects and examining debriefing at different time points.

## Recommendations

Further research is required to better understand beliefs around placebo effects, to develop sensitive ways to inform placebo responders about their treatment allocation and to evaluate the effects of debriefing. This research should combine qualitative research alongside a prospective longitudinal or randomized trial design, using objective outcomes and large clinical samples. Research in this area would increase participant involvement, improve trial methodology and would further our understanding around the therapeutic role of thoughts, feelings and patient-practitioner interactions in RCT's [27].

We recommend that at the informed consent stage investigators ask eligible trial participants whether they would like to be informed about trial results and treatment allocation. It is important to try and avoid creating feelings of embarrassment, mistrust, or disillusionment and to prevent damaging a healing response. For this reason the distinction between 'placebo' and 'placebo effect' should to be made at informed consent, so that participants being enrolled in placebo-controlled trials do not focus on the possibility of receiving a 'dummy' or 'sham' pill, but are informed about the effect that derives from feelings and beliefs, the characteristics of the setting, and the effects of health care interactions [28]. Highlighting this distinction at the informed consent stage should help with debriefing at trial closure.

## Conclusion

Our findings emphasize the importance of involving trial participants at study closure, by sharing study results and individual treatment allocation, while respecting individuals who do not wish to be debriefed. We recommend that this information is communicated in a sensitive manner, perhaps discussing placebo effects within a broader framework by considering the effects of spontaneous recovery, treatment beliefs and health care interactions.

## Competing interests

The author(s) declare that they have no competing interests.

## Authors' contributions

Zelda Di Blasi conceived, conducted and reported the work, and will take responsibility for the integrity of the data and the accuracy of data analysis. All authors gave input to the design of the study and commented on drafts of the paper.

## Pre-publication history

The pre-publication history for this paper can be accessed here:


